# Cholera

**Published:** 1884-08

**Authors:** 


HALL'S
JOURNAL OF HEALTH
—AND—
MEDICINE.

MONTHLY.

E. H. GIBBS, A. M., M. D., EDITOR.

FOUNDED IN 1854.

### CHOLERA.

THERE is one fact in relation to this malady that should be remembered ; it is, that cholera is essentially a filth disease. This by no means implies that its victims are in all cases responsible for the attack. The disease may be forced upon us through the ignorance or negligence of those over whom we have no control, and by avenues that it is not possible to guard, it may enter dwellings where the sanitary conditions seem complete.

On the first appearance of cholera in this country people became panic-stricken, and the pestilence seemed to spread almost without hinderance, until it had carried off large numbers in every condition of life. When the disease had subsided the most careful investigations were made as to its cause, and directions for its treatment in the various stages were widely published. It seems remarkable that beyond the establishment of the "germ" theory, subsequent investigations have added but little to our previous knowledge of the disease or the manner of treating it. Cholera begins in the stomach, where the germs of the disease have entered with the drinking water or are carried down with the food, These germs act only on stomachs that are weakened by disease, excitement or fear. Fear enervates and depresses the vital forces more perhaps than any other ordinary cause. Physicians and nurses seldom fear disease, hence their usual immunity from it.

### *PREVENTIVE MEASURES.*

The most important of these is to examine carefully the water supply. Water from running streams, where the sources have not been contaminated, is always safest ; hence cholera is never so severe in cities thus supplied, as where well water is used. Running water is constantly undergoing purification from the action of the oxygen of the air ; while still water readily receives and holds disease germs that are carried to it in the air or by infiltration through the ground. When cholera is prevalent, all water from open wells or springs, intended for drinking, should be boiled and cooled.

### *HOME SURROUNDINGS.*

Dwellings, outhouses and grounds should be put in complete sanitary condition. Remove all dust, dirt and rubbish from garret to cellar, and from all outbuildings, and burn it. Thoroughly cleanse every spot that has been soiled ; see that no damp places remain in the cellar, for these are hot-beds for disease to propagate ; throw open windows and shutters during several hours each day, and let sunshine and pure air complete the work of purification.

Neglected privy-vaults and cesspools always threaten the health of those who live near them, and if they can not be abated they should be rendered harmless by removal of their contents, or by the free use of disinfectants. The best of all disinfectants is dry earth. If a wagon-load or more is thrown into a vault, it renders it inoffensive, and in a few days the entire contents may be removed and used for fertilizing purposes. After removal sprinkle dry earth in the vault daily, and once a week dissolve half a pound of copperas in a pail of water and sprinkle in the vault.

If the well is within a hundred feet of an old and neglected privy-vault it is almost certain that the water is contaminated. It requires a very careful analysis to detect it, and as the earth between the well and the vault is probably saturated, it will generally be safer to have a new well made at a proper distance.

### *PERSONAL CLEANLINESS.*

The skin is one of the principal avenues through which the waste matters of the body are expelled. It is of the utmost importance that there should be no obstruction to the free exit and prompt removal of this matter. A partial obstruction at once produces fever, and complete closing of the outlet would cause death in a very short time ; hence the importance of keeping the surface of the body cleansed. For this purpose there is nothing better than soap and warm water applied thoroughly twice a week. Water alone is not sufficient to remove the accumulations. Under-clothing should be frequently changed, and outer garments brushed daily to free them from dust. Care should be taken to change the clothing so that it will be adapted to the various changes of temperature. Beds should be aired every day, and there should be free ventilation in sleeping rooms day and night.

### *FOODS.*

The food should be plain but nutritious. Avoid all unripe or over-ripe and wilted fruits and vegetables : these frequently produce cholera-morbus, and such an attack during a season of cholera might act as an exciting cause of cholera ; at any rate, it would produce a depressing effect on the patient through fear of the graver malady.

### *MENTAL EFFECT.*

It seems to be quite generally conceded by physicians who have had experience in the treatment of cholera, that in a large proportion of cases the disease is induced by fever. All strong mental emotions have a debilitating effect on the system, and he who yields to them surrenders easily to the enemy.

### *TREATMENT.*

We often see the statement that persons in the enjoyment of perfect health are stricken with the disease and die within nine or ten hours. We believe this opinion results from the difficulty of obtaining a correct history in such cases. We believe that a diarrhoea lasting *more* than twenty-four hours is an essential preliminary to cholera. After the seeds of the disease have entered the system there are regular stages of the disease to be gone through, if left to itself, and it is certain that more than one day is requisite for its full development. Were this not the case, cholera would be the most terrible of all diseases, instead of one of the most manageable, as it certainly is under proper sanitary conditions and judicious treatment.

Whenever the disease prevails there should be no panic among those whose surroundings are what they should be. The affairs of life should be conducted as usual, but a careful enquiry should be made daily of each child as to any bowel trouble, however slight. These attacks are very insiduous when they are due to incipient cholera, because they are usually painless and hardly attract attention at the time. Looseness of the bowels at such a time is always threatening, and should receive prompt attention. The patient never has felt better perhaps than when cholera is surely commencing its course. Compel the patient to go to bed and lie flat upon his back. Then send at once for a physician. But there are situations where a physician is not quickly to be had, and to wait several hours would be hazardous. We would therefore suggest that it is always well to have an ounce of laudanum on hand, plainly labelled "POISON" and kept ordinarily out of reach of children and careless persons.

Give a child from five to ten years old two drops of laudanum every two hours until the diarrhoea is checked. Give older persons according to age, up to thirty drops. Keep the patient warm and comfortable in bed. Cloths wrung out in warm water or a mustard plaster should be put over the stomach, and a flat-iron heated and wrapped i ı cloths should be put to the feet. If the body or limbs become cold apply mustard plasters freely. The patient should not drink cold water, but in case of considerable thirst a tea made by pouring boiling water on pieces of toasted bread may be taken in moderation. Opium in some form, and calomel, are the specifics for cholera in its first stages, and judiciously administered and with proper nursing every patient will recover in twenty-four hours.

It is only neglected cases that prove fatal among people of good constitutions and good habits. Vomiting and purging are very dangerous symptoms, and when quickly followed by cramp there is not much hope of recovery, as that is often the commencement of fatal collapse.

Cholera is one of those diseases that calls for prompt and heroic treatment at the earliest possible moment. It is like a fire in a building filled with combustible materials. It is easily checked on the start, but soon gains irresittible headway.

In the hands of a physician morphine and calomel given alternately at short intervals and in small doses are the most reliable remedies in any stage of the disease. Hypodermic injections of morphine have been highly recommended, and it would seem that quiuine might be used to advantage in certain cases.

Serious and often fatal complications have followed after all signs of cholera have disappeared. There is reason to believe that these were due to too energetic treatment, and these cases should suggest as great caution in the treatment of cholera as is consistent with the urgency of the symptoms.

